# FlowDock: Geometric Flow Matching for Generative Protein-Ligand Docking and Affinity Prediction

**Published:** 2025-01-15

**Authors:** Alex Morehead, Jianlin Cheng

**Affiliations:** 1 Department of Electrical Engineering & Computer Science, NextGen Precision Health, University of Missouri-Columbia, W1024 Lafferre Hall, 65211, Missouri, USA

**Keywords:** Generative AI model, flow matching, protein-ligand structure, binding affinity

## Abstract

**Motivation:**

Powerful generative AI models of protein-ligand structure have recently been proposed, but few of these methods support both flexible protein-ligand docking and affinity estimation. Of those that do, none can directly model multiple binding ligands concurrently or have been rigorously benchmarked on pharmacologically relevant drug targets, hindering their widespread adoption in drug discovery efforts.

**Results:**

In this work, we propose FlowDock, the first deep geometric generative model based on conditional flow matching that learns to directly map unbound (apo) structures to their bound (holo) counterparts for an arbitrary number of binding ligands. Furthermore, FlowDock provides predicted structural confidence scores and binding affinity values with each of its generated protein-ligand complex structures, enabling fast virtual screening of new (multi-ligand) drug targets. For the well-known PoseBusters Benchmark dataset, FlowDock outperforms single-sequence AlphaFold 3 with a 51% blind docking success rate using unbound (apo) protein input structures and without any information derived from multiple sequence alignments, and for the challenging new DockGen-E dataset, FlowDock outperforms single-sequence AlphaFold 3 and matches single-sequence Chai-1 for binding pocket generalization. Additionally, in the ligand category of the 16th community-wide Critical Assessment of Techniques for Structure Prediction (CASP16), FlowDock ranked among the top-5 methods for pharmacological binding affinity estimation across 140 protein-ligand complexes, demonstrating the efficacy of its learned representations in virtual screening.

**Availability:**

Source code, data, and pre-trained models are available at https://github.com/BioinfoMachineLearning/FlowDock.

## Introduction

Interactions between proteins and small molecules (ligands) drive many of life’s fundamental processes and, as such, are of great interest to biochemists, biologists and drug discoverers. Historically, elucidating the structure, and therefore the function, of such interactions has required that considerable intellectual and financial resources be dedicated to determining the interactions of a single biomolecular complex. For example, techniques such as X-ray diffraction and cryo-electron microscopy have traditionally been effective in biomolecular structure determination, however, resolving even a single biomolecule’s crystal structure can be extremely time and resource-intensive. Recently, new machine learning (ML) methods such as AlphaFold 3 (AF3) [Abramson et al., 2024] have been proposed for directly predicting the structure of an arbitrary biomolecule from its primary sequence, offering the potential to expand our understanding of life’s molecules and their implications in disease, energy research, and beyond.

Although powerful models of general biomolecular structure are compelling, they currently do not provide one with an estimate of the binding affinity of a predicted protein-ligand complex, which may indicate whether a pair of molecules truly bind to each other *in vivo*. It is desirable to predict both the structure of a protein-ligand complex and the binding affinity between them via one single ML system [Dhakal et al., 2022]. Moreover, recent generative models of biomolecular structure are primarily based on noise schedules following Gaussian diffusion model methodology which, albeit a powerful modeling framework, lacks interpretability in the context of biological studies of molecular interactions. In this work, we aim to address these concerns with a new *state-of-the-art* hybrid (structure & affinity prediction) generative model called FlowDock for flow matching-based protein-ligand structure prediction and binding affinity estimation, which allows one to interpretably inspect the model’s structure prediction trajectories to interrogate its common molecular interactions and to screen drug candidates quickly using its predicted binding affinities.

## Related work

### Molecular docking with deep learning.

Over the last few years, deep learning (DL) algorithms (in particular geometric variants) have emerged as a popular methodology for performing end-to-end differentiable molecular docking. Models such as EquiBind [Stärk et al., 2022] and TankBind [Lu et al., 2022] initiated a wave of interest in researching graph-based approaches to modeling protein-ligand interactions, leading to many follow-up works. Important to note is that most of such DL-based docking models were designed to supplement conventional modeling methods for protein-ligand docking such as AutoDock Vina [Eberhardt et al., 2021] which are traditionally slow and computationally expensive to run for many protein-ligand complexes yet can achieve high accuracy with crystal input structures and ground-truth binding pocket annotations.

### Generative biomolecular modeling.

The potential of generative modeling in capturing intricate molecular details in structural biology such as protein-ligand interactions during molecular docking [Corso et al., 2022] has recently become a research focus of ambitious biomolecular modeling efforts such as AF3 [Abramson et al., 2024], with several open-source spin-offs of this algorithm emerging [Discovery et al., 2024, Wohlwend et al., 2024].

### Flow matching.

In the machine learning community, generative modeling with flow matching [Chen and Lipman, 2024, Tong et al., 2024] has recently become an appealing generalization of diffusion generative models [Ho et al., 2020, Karras et al., 2022], enabling one to transport samples between arbitrary distributions for compelling applications in computer vision [Esser et al., 2024], computational biology [Klein et al., 2024], and beyond. As a closely related concurrent work (as our method was developed for the CASP16 competition starting in May 2024 [CASP16-Organizers, 2024]), Corso et al. [2024b] recently introduced and evaluated an unbalanced flow matching procedure for pocket-based flexible docking. However, the authors’ proposed approach mixes diffusion and flow matching noise schedules with geometric product spacesin an unintuitive manner, and neither source code nor data for this work are publicly available for benchmarking comparisons. In Section 3.3, we describe flow matching in detail.

### Contributions.

In light of such prior works, our contributions in this manuscript are as follows:

- We introduce the first simple yet state-of-the-art hybrid generative flow matching model capable of quickly and accurately predicting protein-ligand complex structures and their binding affinities, with source code and model weights freely available.
- We rigorously validate our proposed methodology using standardized benchmarking data for protein-ligand complexes, with our method ranking as a more accurate and generalizable structure predictor than (single-sequence) AF3.
- Our method ranked as a top-5 binding affinity predictor for the 140 pharmaceutically relevant drug targets available in the 2024 community-wide CASP16 ligand prediction competition.
- We release one of the largest ML-ready datasets of apo-to-holo protein structure mappings based on high-accuracy predicted protein structures, which enables training new models on comprehensive biological data for distributional biomolecular structure modeling.

## Methods and materials

The goal of this work is to jointly predict protein-ligand complex structures and their binding affinities with minimal computational overhead to facilitate drug discovery. In Sections 3.1 and 3.2, we briefly outline how FlowDock achieves this and how its key notation is defined. We then describe FlowDock’s training and sampling procedures in Sections 3.3–3.6.

### Overview

**Fig. 1:**
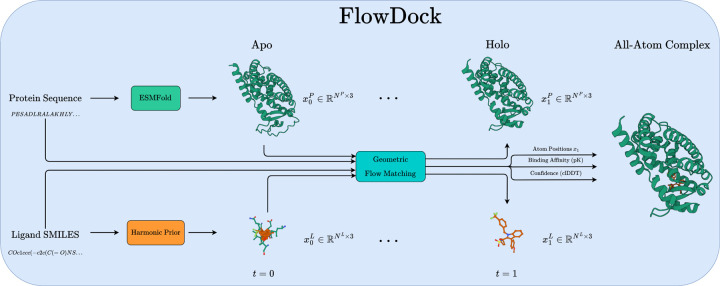
An overview of biomolecular distribution modeling with FlowDock.

Figure 1 illustrates how FlowDock uses geometric flow matching to predict flexible protein-ligand structures and binding affinities. At a high level, FlowDock accepts both (multi-chain) protein sequences and (multi-fragment) ligand SMILES strings as its primary inputs, which it uses to predict an unbound (apo) state of the protein sequences using ESMFold [Lin et al., 2023] and to sample from a harmonic ligand prior distribution [Jing et al., 2024] to initialize the ligand structures using biophysical constraints based on their specified bond graphs. Notably, users can also specify the initial protein structure using one produced by another bespoke method (e.g., AF3 which we use in certain experiments). With these initial structures representing the complex’s state at time t=0, FlowDock employs conditional flow matching to produce fast structure generation trajectories. After running a small number of integration timesteps (e.g., 20 in our experiments), the complex’s state arrives at time t=1, i.e., the model’s estimate of the bound (holo) protein-ligand heavy-atom structure. At this point, FlowDock runs confidence and binding affinity heads to predict structural confidence scores and binding affinities of the predicted complex structure, to rank-order the model’s generated samples.

### Notation

Let $x_{0}$ denote the unbound (apo) state of a protein-ligand complex structure, representing the heavy atoms of the protein and ligand structures as ${\text{x}}_{0}^{P}\in {\mathbb{R}}^{N^{P}\times 3}$ and ${\text{x}}_{0}^{L}\in {\mathbb{R}}^{N^{L}\times 3}$, respectively, where $N^{P}$ and $N^{L}$ are the numbers of protein and ligand heavy atoms. Similarly, we denote the corresponding bound (holo) state of the complex as $x_{1}$. Further, let $s^{P}\in {\left\{1,\ldots ,20\right\}}^{s^{P}}$ denote the type of each amino acid residue in the protein structure, where $S^{P}$ represents the protein’s sequence length. To generate bound (holo) structures, we define a flow model $v_{\theta }$ that integrates the ordinary differential equation (ODE) it defines from time t=0 to t=1.

### Riemannian manifolds and conditional flow matching

In manifold theory, an n-dimensional manifold 𝓜 represents a topological space equivalent to ${\mathbb{R}}^{n}$. In the context of Riemannian manifold theory, each point x∈𝓜 on a Riemannian manifold is associated with a tangent space $T_{x}$. Conveniently, a Riemannian manifold is equipped with a metric $g_{\text{x}}:\;T_{x}𝓜\times T_{x}𝓜\;\to \;\mathbb{R}$ that permits the definition of geometric quantities on the manifold such as distances and geodesics (i.e., shortest paths between two points on the manifold). Subsequently, Riemannian manifolds allow one to define on them probability densities $\int_{𝓜}\rho \left(x\right)dx=1$ where $\rho :𝓜\to {\mathbb{R}}_{+}$ are continuous, non-negative functions. Such probability densities give rise to interpolative probability paths $\rho t:\left[0,1\right]\to \mathbb{P}\left(𝓜\right)$ between probability distributions $\rho_{0},\rho_{1}\in \mathbb{P}\left(𝓜\right)$, where $\mathbb{P}\left(𝓜\right)$ is defined as the space of probability distributions on 𝓜 and the interpolation in probability space between distributions is indexed by the continuous parameter t.

Here, we refer to $\psi_{t}:𝓜\to 𝓜$ as a *flow* on 𝓜. Such a flow serves as a solution to the $\text{ODE}:\frac{d}{dt}\psi_{t}\left(\text{x}\right)=u_{t}\left(\psi_{t}\left(\text{x}\right)\right)$ [Mathieu and Nickel, 2020] which allows one to *push forward* the probability trajectory $\rho_{0}\to \rho_{1}$ to $\rho_{t}$ using $\psi_{t}$ as $\rho_{t}={\left[\psi_{t}\right]}_{\#}\left(\rho_{0}\right)$, with $\psi_{0}\left(x\right)=x$ for $u:\;\left[0,1\right]\times 𝓜\to 𝓜$ (i.e., a smooth time-dependent vector field [Bose et al., 2024]). This insight allows one to perform *flow matching* (FM) [Chen and Lipman, 2024] between $\rho_{0}$ and $\rho_{1}$ by learning a continuous normalizing flow [Papamakarios et al., 2021] to approximate the vector field $u_{t}$ with the parametric $v_{\theta }$. With $\rho_{0}=\rho_{prior}$ and $\rho_{1}=\rho_{data}$, we have that $\rho_{t}$ advantageously permits *simulation-free* training. Although it is not possible to derive a closed form for $u_{t}$ (which generates $\rho_{t}$) with the traditional flow matching (FM) training objective, a *conditional* flow matching (CFM) training objective remains tractable by marginalizing conditional vector fields as $u_{t}\left(\text{x}\right):=\int_{𝓜}u_{t}\left(\text{x}|\text{z}\right)\frac{\rho_{t}\left({\text{x}}_{t}|\text{z}\right)q\left(\text{z}\right)}{\rho_{t}\left(\text{x}\right)}d\text{z}$, where q(z) represents one’s chosen coupling distribution (by default the independent coupling $q\left(\text{z}\right)=q\left({\text{x}}_{0}\right)q\left({\text{x}}_{1}\right))$ between ${\text{x}}_{0}$ and ${\text{x}}_{1}$ via the conditioning variable z. For Riemannian CFM (RCFM) [Chen and Lipman, 2024], the corresponding training objective, with $t∼𝓤\left(0,1\right)$, is:

(1) $$L_{RCFM}\left(\theta \right)=E_{t,q\left(\text{z}\right),\rho_{t}\left({\text{x}}_{t}|\text{z}\right)}{\left\|v_{\theta }\left({\text{x}}_{t},t\right)-u_{t}\left({\text{x}}_{t}|\text{z}\right)\right\|}_{g}^{2},$$

where Tong et al. [2024] have fortuitously shown that the gradients of FM and CFM are identical. As such, to transport samples of the prior distribution $\rho_{0}$ to the target (data) distribution $\rho_{1}$, one can sample from $\rho_{0}$ and use $v_{0}$ to run the corresponding ODE forward in time. In the remainder of this work, we will focus specifically on the 3-manifold ${\mathbb{R}}^{3}$.

### Prior distributions

With flow matching defined, in this section, we describe how we use a bespoke mixture of prior distributions ($\rho_{0}^{P}$ and $\rho_{0}^{L}$) to sample initial (unbound) protein and ligand structures for binding (holo) structure generation targeting our data distribution of crystal protein-ligand complex structures $\rho_{1}$. In Section 4.1, we ablate this mixture to understand its empirical strengths.

#### ESMFold protein prior.

To our best knowledge, FLOWDOCK is among the first methods-concurrently with Corso et al. [2024b]-to explore using structure prediction models with flow matching to represent the unbound state of an arbitrary protein sequence. In contrast to Corso et al. [2024b], we formally define a distribution of unbound (apo) protein structures using the single-sequence ESMFold model as $\rho_{0}^{P}\;\left(x_{0}^{P}\;\right)\;\propto \;\text{ESMFold}\left(s^{P}\;\right)\;+\;\in$, $\in ∼𝓝\left(0,\sigma \right)$, which encourages our model to learn more than a strict mapping between protein apo and holo point masses. Based on previous works developing protein generative models [Dauparas et al., 2022], during training we apply $\in ∼𝓝\left(0,\sigma =1e^{-4}\right)$ to both $x_{0}^{P}$ and $x_{1}^{P}$ to discourage our model from overfitting to computational or experimental noise in its training data. It is important to note that this additive noise for protein structures is not a general substitute for generating a full conformational ensemble of each protein, but to avoid the excessively high computational resource requirements of running protein dynamics methods such as AlphaFlow [Jing et al., 2024] for each protein, we empirically find noised ESMFold structures to be a suitable surrogate.

#### Harmonic ligand prior.

Inspired by the FlowSite model for multi-ligand binding site design [Stark et al., 2024], FlowDock samples initial ligand conformations using a harmonic prior distribution constrained by the bond graph defined by one’s specified ligand SMILES strings. This prior can be sampled as a modified Gaussian distribution via $\rho_{0}^{L}\left({\text{x}}_{0}^{L}\right)\propto \;exp\left(-\frac{1}{2}{\text{x}}_{0}^{L^{T}}\text{L}x_{0}^{L}\right)$ where L denotes a ligand bond graph’s Laplacian matrix defined as L=D−A, with A being the graph’s adjacency matrix and D being its degree matrix. Similarly to our ESMFold protein prior, we subsequently apply $\epsilon ∼𝓝(0,\sigma =1e^{-4})$ to ${\text{x}}_{1}^{L}$ during training.

### Training

We describe FlowDock’s structure parametrization, optimization procedure, and the curation and composition of its new training dataset in the following sections. Further, we provide training and inference pseudocode in Appendix A.

#### Parametrizing protein-ligand complexes with geometric flows.

Based on our experimental observations of the difficulty of scaling up intrinsic generative models [Corso, 2023] that operate on geometric product spaces, FlowDock instead parametrizes 3D protein-ligand complex structures as attributed geometric graphs [Joshi et al., 2023] representing the heavy atoms of each complex’s protein and ligand structures. The main benefit of a heavy atom parametrization is that it can considerably simplify the optimization of a flow model $v_{\theta }$ by allowing one to define its primary loss function as simply as a CondOT path [Pooladian et al., 2023, Jing et al., 2024]:

(2) $$L_{{\mathbb{R}}^{3}}\left(\theta \right)=B_{t,q\left(\text{z}\right),\rho_{t}\left({\text{x}}_{t}|z\right)}{\left\|v_{\theta }\left({\text{x}}_{t},t\right)-{\text{x}}_{1}\right\|}^{2},$$

with the conditional probability path $\rho_{t}$ chosen as

(3) $$\rho_{t}\left(x|z\right)=\rho_{t}\left(\text{x}|{\text{x}}_{0},{\text{x}}_{1}\right)=\left(1-t\right)\cdot {\text{x}}_{0}+t\cdot {\text{x}}_{1},{\text{x}}_{0}∼\rho_{0}\left({\text{x}}_{0}\right)$$

The challenge introduced by this atomic parametrization is that it necessitates the development of an efficient neural architecture that can scalably process all-atom input structures without the exhaustive computational overhead of generative models such as AF3. Fortunately, one such architecture, recently introduced by Qiao et al. [2024] with the NeuralPLexer model, satisfies this requirement. As such, inspired by how the AlphaFlow model was fine-tuned from the base AlphaFold 2 (AF2) architecture using flow matching, to train FlowDock we explored fine-tuning the NeuralPLexer architecture to represent our vector field estimate $v_{\theta }$. Uniquely, we empirically found this idea to work best by fine-tuning the architecture’s score head, which was originally trained with a denoising score matching objective for *diffusion*-based structure sampling, instead using Eqs. 2 and 3. Moreover, we fine-tune all of NeuralPLexer’s remaining intermediate weights and prediction heads including a dedicated confidence head redesigned to predict binding affinities, with the exception of its original confidence head which remains frozen at all points during training.

#### PDBBind-E Data Curation.

To train FlowDock with resolved protein-ligand structures and binding affinities, we prepared PDBBind-E, an enhanced version of the PDBBind 2020-based training dataset proposed by Corso et al. [2024a] for training recent DL docking methods such as DiffDock-L. To curate PDBBind-E, we collected 17,743 crystal complex structures contained in the PDBBind 2020 dataset and 47,183 structures of the Binding MOAD [Hu et al., 2005] dataset splits introduced by Corso et al. [2024a] (n.b., which maintain the validity of our benchmarking results in Section 4 according to time and ligand-based similarity cutoffs) and predicted the structure of these (multi-chain) protein sequences in each dataset split using ESMFold. To optimally align each predicted protein structure with its corresponding crystal structure, we performed a weighted structural alignment optimizing for the distances of the predicted protein residues’ Cα atoms to the crystal heavy atom positions of the complex’s binding ligand, similar to [Corso et al., 2024a]. After dropping complexes for which the crystal structure contained protein sequence gaps caused by unresolved residues, the total number of PDBBind and Binding MOAD predicted complex structures remaining was 17,743 and 46,567, respectively.

#### Generalized unbalanced flow matching.

We empirically observed the challenges of naively training flexible docking models like FlowDock without any adjustments to the sampling of their training data. Accordingly, we concurrently developed a generalized version of *unbalanced* flow matching [Corso et al., 2024b] by defining our coupling distribution $q\left(z\right)$ as

(4) $$q\left({\text{x}}_{0},{\text{x}}_{1}\right)\propto q_{0}\left({\text{x}}_{0}\right)q_{1}\left({\text{x}}_{1}\right)I_{c\left({\text{x}}_{0},{\text{x}}_{1}\right)\in c_{A}},$$

where $c_{A}$ is defined as a set of apo-to-holo assessment filters measuring the structural similarity of the unbound (apo) and bound (holo) protein structures (n.b., not simply their binding pockets) in terms of their root mean square deviation (RMSD) and TM-score [Zhang and Skolnick, 2004] following optimal structural alignment (as used in constructing PDBBind-E). Effectively, we sample independent examples from $q_{0}$ and $q_{1}$ and reject these paired examples if $c({\text{x}}_{0},{\text{x}}_{1})<c_{A_{TM}}$ or *$c({\text{x}}_{0},{\text{x}}_{1})\;\geq c_{A_{RMSD}}$* (n.b., we use $c_{A_{TM}}=0.7$ and $c_{A_{RMSD}}=5Å$ as well as other length-based criteria in our experiments, please see our code for full details).

### Sampling

By default, we apply i=40 timesteps of an Euler solver to integrate FlowDock’s learned ODE $v_{\theta }$ forward in time for binding (holo) structure generation. Specifically, to generate structures, we propose to integrate a Variance Diminishing ODE (VD-ODE) that uses $v_{\theta }$ as

(5) $${\text{x}}_{n+1}=clamp\left(\frac{1-s}{1-t}\cdot \eta \right)\cdot {\text{x}}_{n}+clamp\left(\left(1-\frac{1-s}{1-t}\right)\cdot \eta \right)\cdot v_{\theta }\left({\text{x}}_{n},t\right),$$

where n represents the current integer timestep, allowing us to define $t=\frac{n}{i}$ and $s=\frac{n+1}{i}$ ; η=1.0 in our experiments; and *clamp* ensures both the LHS and RHS of Eq. 5 are lower and upper bounded by $1e^{-6}$ and $1-1e^{-6}$, respectively. We experimented with different values of η yet ultimately settled on 1.0 since this yielded FlowDock’s best performance for structure and affinity prediction. Intuitively, this VD-ODE solver limits the high levels of variance present in the model’s predictions $v_{\theta }$ during early timesteps by sharply interpolating towards $v_{\theta }$ in later timesteps.

## Results

### PoseBench Protein-Ligand Docking

#### PoseBusters Benchmark set.

**Fig. 2:**
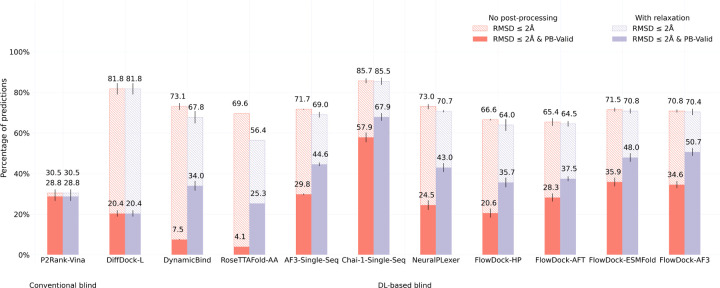
Protein-ligand docking success rates of each baseline method on the PoseBusters Benchmark set (n=308). Error bars: 3 runs.

**Fig. 3:**
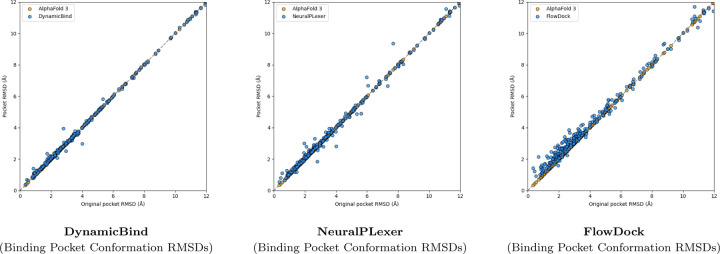
Comparison of each flexible docking method’s protein conformational changes made for the PoseBusters Benchmark set (n=308).

In Figures 2 and 3, we illustrate the performance of each baseline method for protein-ligand docking and protein conformational modification with the commonly-used PoseBusters Benchmark set [Buttenschoen et al., 2024], provided by version 0.6.0 the PoseBench protein-ligand benchmarking suite [Morehead et al., 2024], which consists of 308 distinct protein-ligand complexes released after 2020. It is important to note that this benchmarking set can be considered a moderately difficult challenge for methods trained on recent collections of data derived from the Protein Data Bank (PDB) [Bank, 1971] such as PDBBind 2020 [Liu et al., 2015], as all of these 308 protein-ligand complexes are not contained in the most common training splits of such PDB-based data collections [Buttenschoen et al., 2024] (with the exception of AF3 which uses a cutoff date of September 30, 2021). Moreover, as described by Buttenschoen et al. [2024], a subset of these complexes also have very low protein sequence similarity to such training splits.

Figure 2 shows that FlowDock consistently improves over the original NeuralPLexer model’s docking success rate in terms of its structural and chemical accuracy (as measured by the RMSD ≤ 2Å & PB-Valid metric [Buttenschoen et al., 2024]) and inter-run stability (as measured by the error bars listed). Notably, FlowDock achieves a 10% higher docking success rate than NeuralPLexer without any structural energy minimization driven by molecular dynamics software [Eastman et al., 2017], and with energy minimization its docking success rate increases to 51%, outperforming single-sequence AF3 and achieving second-best performance on this dataset compared to single-sequence Chai-1 [Discovery et al., 2024]. Important to note is that Chai-1, like AF3, is a 10x larger model trained for one month using 128 NVIDIA A100 80GB GPUs on more than twice as much data in the PDB deposited up to 2021, whereas FlowDock is trained using only 4 80GB H100 GPUs for one week, representing a 32x reduction in GPU hours required for training. Additionally, FlowDock outperforms the *hybrid* flexible docking method DynamicBind [Lu et al., 2024] by more than 16%, which is a comparable model in terms of its size, training, and downstream capabilities for drug discovery. Our results with ablated versions of FlowDock trained instead with a protein harmonic prior (FlowDock-HP) or with affinity prediction frozen until a fine-tuning phase (FlowDock-AFT) highlight that the protein ESMFold prior the base FlowDock model employs has imbued it with meaningful structural representations for accurate ligand binding structure prediction that are robust to changes in the source method of FlowDock’s predicted protein input structures (e.g., FlowDock-ESMFold vs. FlowDock-AF3), providing users with multiple structure prediction options (e.g., ESMFold for faster and commercially available prediction inputs).

A surprising finding illustrated in Figure 3 is that no method can consistently improve the binding pocket RMSD of AF3’s initial protein structural conformations, which contrasts with the results originally reported for flexible docking methods such as DynamicBind which used structures predicted by AF2 [Jumper et al., 2021] in its experiments. From this figure, we observe that DynamicBind and NeuralPLexer both infrequently modify AF3’s initial binding pocket structure, whereas FlowDock often modifies the pocket structure during ligand binding. The former two methods occasionally improve largely-correct initial pocket conformations by ∼1Å, whereas FlowDock primarily does so for mostly-incorrect initial pockets.

#### DockGen-E set.

**Fig. 4:**
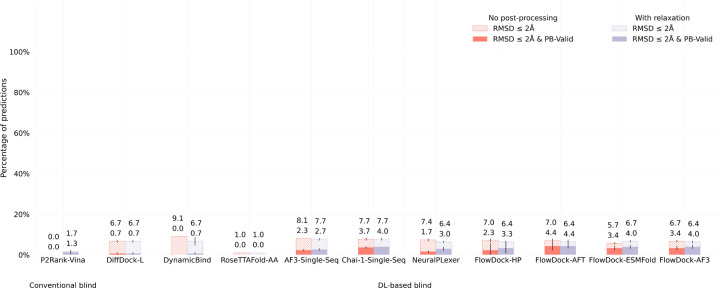
Protein-ligand docking success rates of each baseline method on the DockGen-E set (n=122). Error bars: 3 runs.

**Fig. 5:**
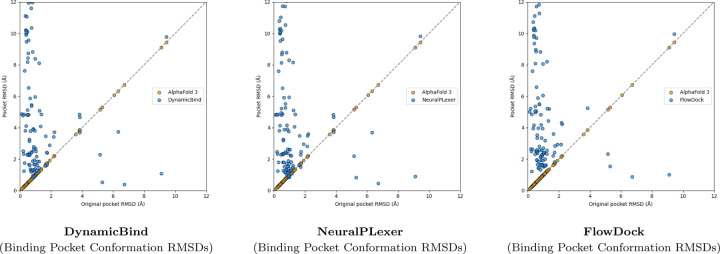
Comparison of each flexible docking method’s protein conformational changes made for the DockGen-E set (n=122).

To assess the generalization capabilities of each baseline method, in Figures 4 and 5, we report each method’s protein-ligand docking and protein conformational modification performance for the novel (i.e., naturally rare) protein binding pockets found in the new DockGen-E dataset from PoseBench. Each of DockGen-E’s protein-ligand complexes represents a distinct binding pocket that facilitates a unique biological function described by its associated ECOD domain identifier [Corso et al., 2024a]. As our results for the DockGen-E dataset show in Figure 4, most DL-based docking or structure prediction methods have likely not been trained or overfitted to these binding pockets, as this dataset’s best docking success rate achieved by any method is approximately 4%, much lower than the 68% best docking success rate achieved for the PoseBusters Benchmark set. We find further support for this phenomenon in Figure 5, where we see that all DL-based flexible docking methods find it challenging to avoid degrading the initial binding pocket state predicted by AF3 yet all methods can *restore* a handful of AF3 binding pockets to their bound (holo) form. This suggests that all DL methods (some more so than others) struggle to generalize to novel binding pockets, yet FlowDock achieves top performance in this regard by tying with single-sequence Chai-1. To address this generalization issue, in future work, training new DL methods on diverse (e.g., PLINDER [Durairaj et al., 2024]) binding pockets may be necessary.

#### Computational resources.

To formally measure the computational resources required to run each baseline method, in Table 1 we list the average runtime (in seconds) and peak CPU (GPU) memory usage (in GB) consumed by each method when running them on a 25% subset of the Astex Diverse dataset [Hartshorn et al., 2007] (baseline results taken from Morehead et al. [2024]). Here, we notably find that FlowDock provides the second lowest computational runtime and GPU memory usage compared to all other DL methods, enabling one to use commodity computing hardware to quickly screen new drug candidates using combinations of FlowDock’s predicted heavy-atom structures, confidence scores, and binding affinities.

### PDBBind Binding Affinity Estimation

In this section, we explore binding affinity estimation with FlowDock using the PDBBind 2020 test dataset (n=363) originally curated by [Stärk et al., 2022], with benchmarking results shown in Table 2. Popular affinity prediction baselines listed in Table 2 such as TankBind [Lu et al., 2022] and DynamicBind [Lu et al., 2024] demonstrate that accurate affinity estimations are possible using hybrid DL models of protein-ligand structures and affinities. Here, we find that, as a hybrid deep generative model, FlowDock provides the best Pearson and Spearman’s correlations compared to all other baselines including FlowDock-HP (a fully harmonic variant of FlowDock) and FlowDock-AFT (an ESMFold prior variant trained first for structure prediction and then with affinity fine-tuning) and produces compelling root mean squared error (RMSE) and mean absolute error (MAE) rates compared to the previous state-of-the-art method DynamicBind. Referencing Table 1, we further note that FlowDock’s average computational runtime per protein-ligand complex is more than 3 times lower than that of DynamicBind, demonstrating that FlowDock, to our best knowledge, is currently the *fastest* binding affinity estimation method to match or exceed DynamicBind’s level of accuracy for predicting binding affinities using the PDBBind 2020 dataset.

**Fig. 6:**
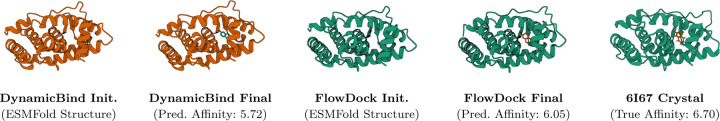
Comparison of DynamicBind and FlowDock’s predicted structures (w/o hydrogens) and crystal PDBBind test example 6I67.

In Figure 6, we provide an illustrative example of a protein-ligand complex in the PDBBind test set (6I67) for which FlowDock predicts notably more accurate complex structural motions and binding affinity values than the hybrid DL method DynamicBind, importantly recognizing that the right-most protein loop domain should be moved further to the right to facilitate ligand binding. One should note that, for historical reasons, our experiments with this PDBBind-based test set employed protein structures predicted by ESMFold (not AF3). In the next section, we explore an even more practical application of FlowDock’s fast and accurate structure and binding affinity predictions in the CASP16 ligand prediction competition.

### CASP16 Protein-Ligand Binding Affinity Prediction

**Fig. 7:**
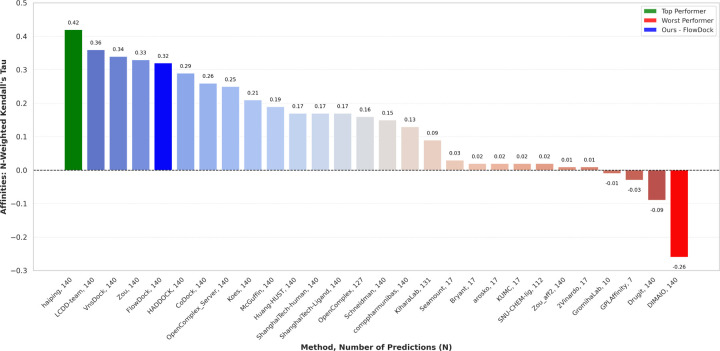
Protein-ligand binding affinity prediction rankings for the CASP16 ligand prediction category (n=140).

In Figure 7, we illustrate the performance of each predictor group for blind protein-ligand binding affinity prediction in the ligand category of the CASP16 competition held in summer 2024, in which pharmaceutically relevant binding ligands were the primary focus of this competition. Notably, FlowDock is the *only* hybrid (structure & affinity prediction) ML method represented among the top-5 predictors, demonstrating the robustness of its knowledge of protein-ligand interactions. Namely, all other top prediction methods were trained specifically for binding affinity estimation assuming a predicted or crystal complex structure is provided. In contrast, in CASP16, we demonstrated the potential of using FlowDock to predict *both* protein-ligand structures and binding affinities and using its top-5 predicted structures’ structural confidence scores to rank-order its top-5 binding affinity predictions. Ranked 5th for binding affinity estimation, these results of the CASP16 competition demonstrate that this dual approach of predicting protein-ligand structures and binding affinities with a single DL model (FlowDock) yields compelling performance for virtual screening of pharmaceutically interesting molecular compounds.

## Conclusion

In this work, we have presented FlowDock, a novel, state-of-the-art deep generative flow model for fast and accurate (hybrid) protein-ligand binding structure and affinity prediction. Benchmarking results suggest that FlowDock achieves structure prediction results better than single-sequence AF3 and comparable to single-sequence Chai-1 and outperforms existing hybrid models like DynamicBind across a range of binding ligands. Lastly, we have demonstrated the pharmaceutical virtual screening potential of FlowDock in the CASP16 ligand prediction competition, where it achieved top-5 performance. Future work could include retraining the model on larger and more diverse clusters of protein-ligand complexes, experimenting with new ODE solvers, or scaling up its parameter count to see if it displays any scaling law behavior for structure or affinity prediction. As a deep generative model for structural biology made available under an MIT license, we believe FlowDock takes a notable step forward towards fast, accurate, and broadly applicable modeling of protein-ligand interactions.

## Figures and Tables

**Table 1.** Computational resource requirements. The average structure prediction runtime (in seconds) and peak memory usage (in GB) of baseline methods on a 25% subset of the Astex Diverse dataset [Hartshorn et al., 2007] using an NVIDIA 80GB A100 GPU for benchmarking (with baselines taken from [Morehead et al., 2024]). The symbol - denotes a result that could not be estimated.

Method	Runtime (s)	CPU Memory Usage (GB)	GPU Memory Usage (GB)
P2Rank-Vina	1,283.70	9.62	0.00
DiffDock-L	88.33	8.99	70.42
DynamicBind	146.99	5.26	18.91
RoseTTAFold-All-Atom	3,443.63	55.75	72.79
AF3	3,049.41	-	-
AF3-Single-Seq	58.72	-	-
Chai-1-Single-Seq	114.86	58.49	56.21
NeuralPLexer	29.10	11.19	31.00
**FlowDock**	39.34	11.98	25.61

**Table 2.** Binding affinity estimation using PDBBind test set. For all methods, binding affinities were predicted in *one shot* using the commonly-used 363 PDBBind (ligand and time-split) test complexes (with splits and baselines from Lu et al. [2024]). Results for FlowDock are reported as the mean and standard error of measurement (*n* = 3) of each metric over three independent runs. Note that, for historical reasons, the results for each version of FlowDock were obtained using ESMFold predicted protein input structures.

Method	Pearson (↑)	Spearman (↑)	RMSE (↓)	MAE (↓)
GIGN	0.286	0.318	1.736	1.330
TransformerCPI	0.470	0.480	1.643	1.317
MONN	0.545	0.535	1.371	1.103
TankBind	0.597	0.610	1.436	1.119
DynamicBind (One-Shot)	0.665	0.634	1.301	1.060
FlowDock-HP	0.577 ± 0.001	0.560 ± 0.001	1.516 ± 0.001	1.196 ± 0.002
FlowDock-AFT	0.663 ± 0.003	0.624 ± 0.003	1.392 ± 0.005	1.113 ± 0.003
**FlowDock**	**0**.**705** ± **0**.**001**	**0**.**674** ± **0**.**002**	1.363±0.003	1.067±0.003

## Geometric Flow Matching Training and Inference

We characterize FlowDock’s training and sampling procedures in Sections 3.5 and 3.6 of the main text, respectively. To further illustrate how training and inference with FlowDock work, in Algorithms 1 and 2 we provide the corresponding pseudocode. For more details, please see our accompanying source code.

$$\begin{array}{l} \bar{\underline{\text{Algorithm}\;\text{1}\;\text{Training}\;\;\;\;\;\;\;\;\;\;\;\;\;\;\;\;\;\;\;\;\;\;\;\;\;\;\;\;\;\;\;\;\;\;\;\;\;}}\\ \text{Require}:\text{Training examples of binding site-aligned apo}\left(\text{holo}\right)\\ \;\text{protein }\left(\text{ligand}\right)\;\text{structures, protein sequences, ligand SMILES}\\ \;\text{strings, and binding affinities}\;\left\{\left(X_{a_{i}}^{P},X_{h_{i}}^{P},X_{h_{i}}^{L},S_{i},M_{i},B_{i}\right)\right\}\\ \;1:\;\text{for}\;\text{all}\;\left(X_{a_{i}}^{P},X_{h_{i}}^{P},X_{h_{i}}^{L},S_{i},M_{i},B_{i}\right)\;\text{do}\\ \;2:\;\text{Extract}\;x_{1}^{P},x_{1}^{L}\leftarrow \text{HeavyAtoms}\left(X_{h_{i}}^{P},X_{h_{i}}^{L}\right)\\ \;3:\;\text{Sample}\;x_{0}^{P}\leftarrow \text{ESMFold}\left(S_{i}\right)+\in ,\;\in ∼𝓝\left(0,\sigma =1e^{-4}\right)\\ \;4:\;\text{Sample}\;x_{0}^{L}\leftarrow \text{HarmonicPrior}\left(M_{i_{frag}}\right),\;\forall frag\in M_{i}\\ \;5:\;\text{Sample}\;t∼𝓤\left(0,1\right)\\ \;6:\;\text{Concatenate}\;x_{0}=\text{Concat}\left(x_{0}^{P},x_{0}^{L}\right)\\ \;7:\;\text{Concatenate}\;x_{1}=\text{Concat}\left(x_{1}^{P},x_{1}^{L}\right)\\ \;8:\;\text{Interpolate}\;x_{t}\leftarrow t\cdot x_{1}+\left(1-t\right)\cdot x_{0}\\ \;9:\;\text{Predict}\;{\hat{X}}_{h_{i}}\leftarrow \text{NeuralPLexer}\left(S_{i},M_{i,}x_{t},t\right)\\ 10:\;\text{Predict}\;{\hat{B}}_{i}\leftarrow {\text{ESDM}}_{aff}\left(S_{i},M_{i},\text{StopGrad}\left({\hat{X}}_{h_{i}}\right)\right)\\ 11:\;\text{Optimize losses}\;{ℒ}_{X}:{\text{λ}}_{X}\cdot \text{FAPE}\left(X_{h_{i},}{\hat{X}}_{h_{i}}\right)+{ℒ}_{B}:={\text{λ}}_{B}\cdot \\ \;\;\text{MSE}\left({\hat{B}}_{i},B_{i}\right),\;\lambda_{X}=0.2,\;\lambda_{B}=0.1\\ \underline{12:\;\text{end}\;\text{for}\;\;\;\;\;\;\;\;\;\;\;\;\;\;\;\;\;\;\;\;\;\;\;\;\;\;\;\;\;\;\;\;\;\;\;\;\;\;\;\;\;\;\;\;\;\;\;\;} \end{array}$$

$$\begin{array}{l} \bar{\underline{\text{Algorithm}\;\text{2}\;\text{Inference}\;\;\;\;\;\;\;\;\;\;\;\;\;\;\;\;\;\;\;\;\;\;\;\;\;\;\;\;\;\;\;\;\;\;\;\;\;\;\;\;\;\;\;\;\;\;\;\;\;\;\;\;\;\;\;\;\;\;\;\;\;\;\;\;\;\;\;\;\;\;\;\;\;\;\;\;\;\;}}\\ \text{Require}:\text{Protein sequences and ligand SMILES strings}\left(S,M\right)\\ \text{Ensure:}\;\text{Sampled top-5 heavy-atom structures}\;\hat{X}\;\text{with confidence}\\ \;\;\;\text{scores}\;\hat{C}\;\text{and}\;\text{binding}\;\text{affinities}\;\hat{B}\\ \;1:\text{Sample}\;x_{0}^{P}\leftarrow \text{ESMFold}\left(S\right)+\in ,\;\in ∼𝓝\left(0,\sigma =1e^{-4}\right)\\ \;2:\;\text{Sample}\;x_{0}^{L}\leftarrow \text{HarmonicPrior}\left(M_{fragg}\right),\;\forall frag\in M\\ \;3:\text{Concatenate}\;x_{0}=\text{Concat}\left(x_{0}^{P},x_{0}^{L}\right)\\ \;4:\text{for}\;n\leftarrow 0\;i\;\text{do}\\ \;5:\;\;\text{Let}\;t\leftarrow \frac{n}{i}\;\text{and}\;s\leftarrow \frac{n+1}{i}\\ \;6:\;\;\text{Predict}\;\hat{X}\leftarrow \text{NeuralPLexer}\left(S,M,x_{n},t\right)\;\\ \;7:\;\;\text{if}\;n=i-1\;\text{then}\\ \;8:\;\;\;\text{Predict}\;\hat{C}\leftarrow {\text{ESDM}}_{conf}\left(S,M,\hat{X}\right)\;\#\text{Pre-trained}\\ \;9:\;\;\;\;\text{Predict}\hat{B}\leftarrow {\text{ESDM}}_{aff}\left(S,M,\hat{X}\right)\\ 10:\;\;\;\text{Rank}\;\text{top}-5\;\hat{X}\text{and}\;\hat{B}\;\text{using}\;\hat{C}\\ 11:\;\;\;\text{return}\;\hat{X},\hat{C},\hat{B}\\ 12:\;\;\text{end}\;\text{if}\\ 13:\;\;\text{Extract}\;{\hat{x}}_{1}\leftarrow \text{HeavyAtoms}\left(\hat{X}\right)\\ 14:\;\;\text{Align}\;x_{n}\leftarrow \text{RMSDAlign}\left(x_{n},{\hat{x}}_{1}\right)\\ 15:\;\;\text{Interpolate}\;x_{n+1}=clamp\left(\frac{1-s}{1-t}\cdot \eta \right)\cdot x_{n}+clamp\left(\left(1-\frac{1-s}{1-t}\right)\cdot \eta \right)\cdot {\hat{x}}_{1},\;\eta =1\\ \underline{16.\;\text{end}\;\text{for}\;\;\;\;\;\;\;\;\;\;\;\;\;\;\;\;\;\;\;\;\;\;\;\;\;\;\;\;\;\;\;\;\;\;\;\;\;\;\;\;\;\;\;\;\;\;\;\;\;\;\;\;\;\;\;\;\;\;\;\;\;\;\;\;\;\;\;\;\;\;\;\;\;\;\;\;\;\;\;\;\;\;\;\;\;\;\;\;} \end{array}$$
